# Hierarchical Bayesian inference for ion channel screening dose-response data

**DOI:** 10.12688/wellcomeopenres.9945.2

**Published:** 2017-03-13

**Authors:** Ross H Johnstone, Rémi Bardenet, David J Gavaghan, Gary R Mirams

**Affiliations:** 1 Computational Biology, Department of Computer Science, University of Oxford, Oxford, UK; 2 CNRS & CRIStAL, Université de Lille, Lille, France; 3 Centre for Mathematical Medicine & Biology, School of Mathematical Sciences, University of Nottingham, Nottingham, UK

**Keywords:** dose-response, concentration-effect, Hill curve, IC50, parameter fitting, Bayesian inference, hierarchical

## Abstract

Dose-response (or ‘concentration-effect’) relationships commonly occur in biological and pharmacological systems and are well characterised by Hill curves. These curves are described by an equation with two parameters: the inhibitory concentration 50% (IC50); and the Hill coefficient. Typically just the ‘best fit’ parameter values are reported in the literature. Here we introduce a Python-based software tool, ***PyHillFit*** , and describe the underlying Bayesian inference methods that it uses, to infer probability distributions for these parameters as well as the level of experimental observation noise. The tool also allows for hierarchical fitting, characterising the effect of inter-experiment variability. We demonstrate the use of the tool on a recently published dataset on multiple ion channel inhibition by multiple drug compounds. We compare the maximum likelihood, Bayesian and hierarchical Bayesian approaches. We then show how uncertainty in dose-response inputs can be characterised and propagated into a cardiac action potential simulation to give a probability distribution on model outputs.

## 1 Introduction

In this article, we describe the approach our software tool takes to inferring parameters of dose-response curves from experimental data. This introduction addresses the problem, standard approach, and our motivation for developing an approach that also characterises uncertainty in dose-response parameters, due to variability in the data.

### 1.1 Dose-response curves

**Figure 1.**
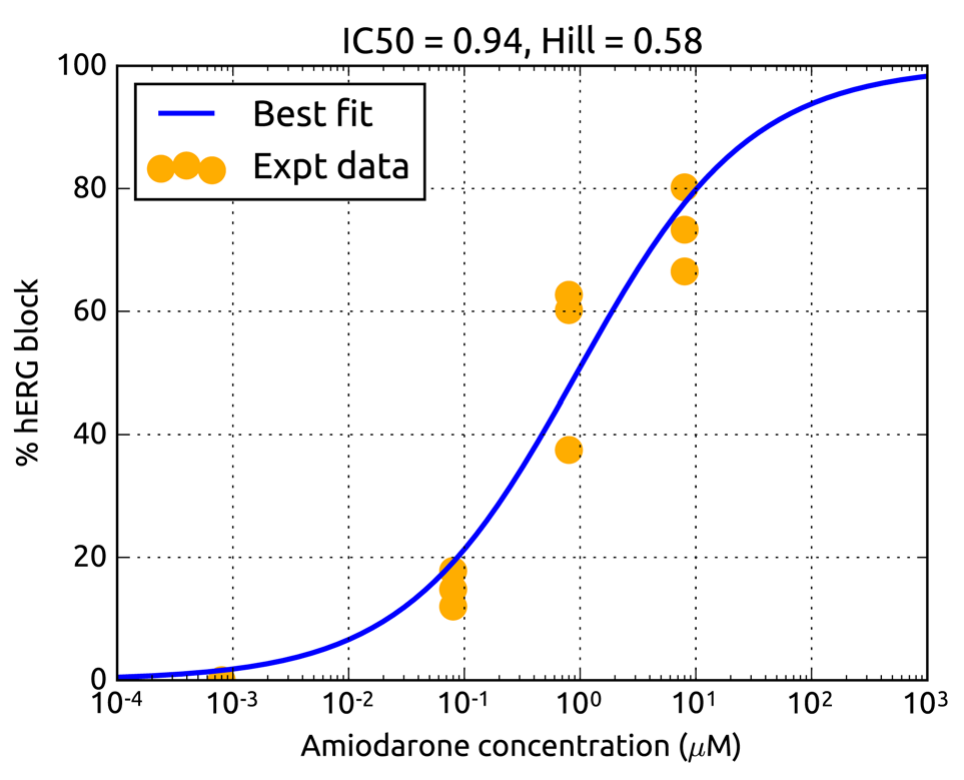
A dose-response curve fitted to experimental data for hERG block by amiodarone from the Crumb *et al.* (2016) dataset. A dose-response curve is shown fitted to all data points at once with a least-square-differences algorithm, with the resulting parameters shown at the top of the graph.

‘Dose-response’ (or ‘concentration-effect’) curve-fitting is generally performed to describe how increasing compound concentration provokes a response in a process. ‘Dose-response’ generally relates to *in-vivo* experiments where a drug dose is administered but the concentration at the relevant site is not precisely known; whereas ‘concentration-effect’ generally relates to *in-vitro* experiments where the concentration is accurately applied. We will refer to both as ‘dose-response’ in this article for simplicity. In the case study we will pursue, the ‘response’ is binding and blocking of ion channels, measured via inhibition of ion currents. Dose-response curves are summarised by two parameters: an inhibitory concentration 50% (IC50) value, that is the concentration of the compound that gives 50% of the maximum effect; and a Hill coefficient, which sets the ‘steepness’ of the curve as it passes the IC50. Examples of dose-response data and a fitted curve are given in Figure 1.

The equation for a dose-response curve was proposed by Hill (1910), and subsequently another name for the curve is a *Hill curve* ( Weiss, 1997). If we let *x* be the concentration of a compound, we describe the effect of the compound by

$$\text{response}=f_{DR}\left(x;IC50,Hill\right):=\frac{100}{1+{\left(\frac{IC50}{x}\right)}^{Hill}},\;(1)$$

where IC50 and *Hill* are parameters that take positive values. In our motivating example this response will be “% block” of a particular type of ion channel.

### 1.2 Standard fitting procedure

A Hill curve is often fitted to all data points simultaneously, to obtain ‘average’ IC50 values and Hill coefficients for a particular curve. This gives the most likely set of parameter values. For example, Crumb *et al.* (2016) recently published dose-response screening data for 30 compounds on 7 different ion channels, along with best-fit IC50 values and Hill coefficients. But taking this approach, there is no associated probability given to these IC50 and Hill values; different possible ranges for these parameter values are not considered. The usual fitting procedure can also give rise to models which differ in behaviour from each individual experiment, as shown by Pathmanathan *et al.* (2015) in the case of inactivation of the fast sodium current in action potential models, and as we will show in the case studies below.

### 1.3 Variability and uncertainty

Real-world experiments exhibit *intrinsic* and *extrinsic* variability. The characterisation of this variability is becoming of greater importance as we move to quantitatively predictive models, particularly as part of the global cardiac modelling effort ( Johnstone *et al.*, 2016a; Mirams *et al.*, 2016; Pathmanathan *et al.*, 2015). Intrinsic variability describes fluctuations that may be due to inherent randomness, and extrinsic variability describes differences between individuals (in this case cells/experiments). Variability contributes to uncertainty, but there are other sources of uncertainty when modelling and performing experiments ( Vernon *et al.* (2010) provide a good introduction). There will also be observation error, which arises from imperfect measurements, thus introducing more uncertainty.

If we are going to use a model to predict future behaviour, or infer some underlying behaviour, we want to study the impact of uncertainty and give probabilistic predictions. Here, if there is a distribution of parameter values that could have given rise to the experimental dose-response data, we would like to capture this distribution ( *uncertainty characterisation*). When using these data as inputs to further simulations (as discussed below in Section 6) we would then construct a distribution of possible outputs corresponding to the distribution of inputs, a process known as *uncertainty propagation*. The whole process is known as *uncertainty quantification*, or *UQ* ( US National Research Council, 2012), and is part of a framework for ensuring safety-critical simulations are reliable which is known as validation, verification and uncertainty quantification, or *VVUQ* ( Pathmanathan & Gray, 2013).

### 1.4 Motivation

As part of the *Comprehensive in vitro Pro-arrhythmia Assay* initiative (CiPA, Fermini *et al.*, 2016; Sager *et al.*, 2014), it is proposed that pharmaceutical compounds be tested on up to 7 ion channels that both strongly influence ventricular repolarisation and are frequently blocked by pharmaceutical compounds. The proposal is for ion channel screening data to be passed into an *in silico* human ventricular action potential model to see if the compound produces pro-arrhythmic behaviour, or indicators of such behaviour, at the whole-cell level.

In this article we present a software tool to infer distributions of possible dose-response curves from experimental data. When making predictions of block at a given concentration, these distributions of possible dose-response curves provide us with a probability distribution for the level of block at that concentration. To illustrate the consequences of this, we use the distributions of block (for multiple ion channels) inferred from data provided in Crumb *et al.* (2016) as inputs into an *in silico* action potential model. We then run forward simulations to predict a distribution of outputs — in this case action potential durations resulting from application of a compound at a particular concentration. We show that given the limited number of repeats of ion-channel experiments, there are wide ranges of predicted action potentials, with overlapping results for different compounds with different associated pro-arrhythmic risks.

### 1.5 Our approach

To explore and characterise the uncertainty in the dose-response measurements published by Crumb *et al.*, and to propagate these uncertainties into model predictions, we use a Bayesian statistical framework to explore different possible dose-response curves that may have produced these data. Each dose-response curve is assigned a likelihood score, which, roughly speaking, describes how well the curve fits the data. Instead of computing point-estimates for the IC50 value and Hill coefficient, we infer probability distributions of these parameters, as well as a distribution for the possible observational noise. This provides us with a method for propagating uncertainty in experimental data into simulations by drawing parameters from these inferred distributions, and using these samples as simulation inputs.

We describe two different types of Bayesian statistical models: one where all data points are treated equally (as though they were obtained from the same experiment); and another where we believe that each repeat of an experiment has distinct properties (through some source of inter-experiment variability) and therefore its own set of parameters to infer. The first case we will refer to as ‘single-level’, and the second case as ‘hierarchical’. The single-level case does not consider extrinsic variability, since we are assuming that all data points are generated by the same behaviour. The hierarchical case does consider extrinsic variability, which we model by assuming that each experimental dataset was generated according to its own IC50 value and Hill coefficient, which may vary across experiments.

## 2 Bayesian statistical modelling approach

We use a Bayesian framework to quantify the uncertainty present in the ion channel screening data. The tool reads in doses in μM, but instead of working with IC50 in μM, we work with pIC50, where

$$pIC50\text{ߙ}[\text{log}\left(\text{Molar}\right)]=6-{\text{log}}_{10}\left(IC50\left[\mu \text{Molar}\right]\right),\;(2)$$

with square brackets indicating units. This transformation makes it much easier for fitting algorithms to explore the parameter space, as linear variation in IC50s does not result in linear changes to dose-response curves, which are commonly plotted on log scales. The dose-response model we therefore work with in practice is

$$\%\;\text{channel}\;\text{block}=f\left(x;pIC50,Hill\right):=f_{DR}\left(x;10^{\left(6-pIC50\right)},Hill\right).\;(3)$$

So we assume that the underlying behaviour is described by the dose-response model, *f*, given by Equation (3). We assume that an experimental observation is Normally distributed around some underlying behaviour with some standard deviation, σ (that has the same units as the measured response). That is, given an applied compound concentration, *x*, our statistical model is that the response, *y*, is a Normally-distributed random variable with mean *f*( *x*; *pIC*50, *Hill*) and standard deviation *σ*, that is:

$$y∼\left(f\left(x;pIC50,Hill\right),\sigma^{2}\right).\;(4)$$

When we have noisy data, different sets of parameters might allow us to fit the equation to the experimental data equally well. In our Bayesian framework, we treat these model parameters as random variables, in part due to the uncertainty introduced through observational error and any parameter identifiability problems (see e.g. Daly *et al.* (2015); Raue *et al.* (2009); Siekmann *et al.* (2012) for a discussion of how identifiability relates to inferred probability distributions). We therefore want to infer a probability distribution, instead of point-estimates, for the parameters *pIC*50, *Hill* and *σ*. This probability distribution, *p*( *θ*| *data*), is the *posterior* distribution of the parameters, *θ*, given the observed experimental data.

The posterior distribution is defined using Bayes’ Theorem:

$$p\left(\theta |data\right)=\frac{p\left(data|\theta \right)p\left(\theta \right)}{\int_{\theta }p\left(data|\theta \right)p\left(\theta \right)\text{d}\theta },\;(5)$$

where *p*( *data*| *θ*) is the likelihood of the parameters *θ* under our model given the observed data **y**, and *p*( *θ*) is the *prior* distribution of the parameters *θ*. The prior distribution contains our prior knowledge or belief about the parameters before observing any data.

The integral in the denominator of Equation (5) is generally intractable, so we use Markov chain Monte Carlo (MCMC) methods to approximate *p*( *θ*| *data*). MCMC methods only require that we can evaluate the posterior distribution, pointwise, up to a factor of a constant, so it is enough to have that

p(θ|data)∝p(data|θ)p(θ),(6)

to allow us to construct an approximation to the posterior distribution.

## 3 Single-level model

In our example, each experiment consisted of applying one or more concentrations of a compound to a cell and measuring the degree of block of an ion current. There were multiple recordings, leading to multiple response data-points at each concentration.

### 3.1 Methods

**Figure 2.**
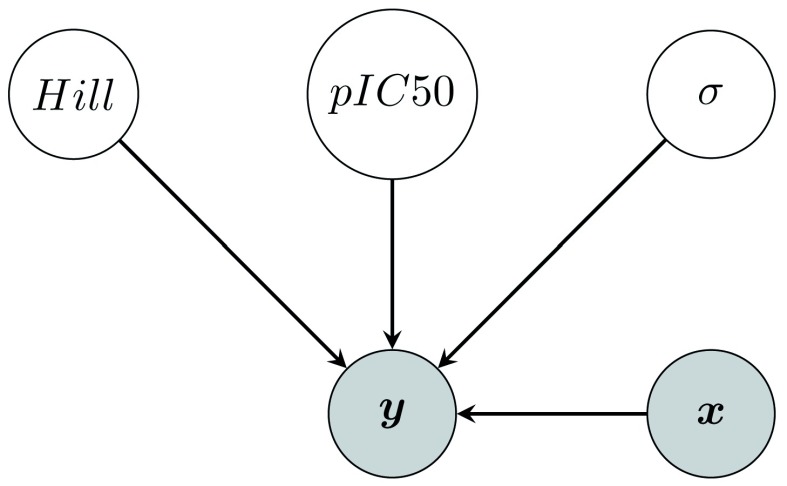
Statistical model for the generation of dose-response data *y*. All non-shaded variables are parameters for which we wish to infer probability distributions. **y** = { *y* ^(1)^, …, *y* ^( *K*)^} is the vector of all experimentally-recorded response data points.

In this statistical model, we will assume that there is no inter-experiment variability, so all data points are (effectively) from one experiment, and all the data points are generated using the same set of parameter values. Under this model, the data for hERG block by amiodarone ( Crumb *et al.*, 2016), for example, is generated by the process schematic shown in Figure 2.

Under our statistical model shown in Figure 2, all data points *y* ^( *j*)^ from a single drug and channel combination are identically independently distributed according to

$$y^{\left(j\right)}∼\left(f\left(x^{\left(j\right)};pIC50,Hill\right),\sigma^{2}\right),\;(7)$$

where *x* ^( *j*)^ is the applied compound concentration, and *j* = 1, …, *K*, where *K* is the total number of data points. The likelihood of the parameters *pIC*50, *Hill*, and *σ*, given a single data point *y* ^( *j*)^ is then

$$p\left(y^{\left(j\right)}|pIC50,Hill,\sigma \right)=\frac{1}{\sqrt{2\pi \sigma^{2}}}\text{exp}\left(-\frac{{\left(y^{\left(j\right)}-f\left(x^{\left(j\right)};pIC50,Hill\right)\right)}^{2}}{2\sigma^{2}}\right).\;(8)$$

In practice, we work with the log of the target distribution, and therefore the log of the likelihood given in Equation 8. For more details on the implementation, see Johnstone *et al.*, (2016a, Supplement B2).

In the data published by Crumb *et al.*, any observations below 0% or above 100% were capped to 0% or 100%, respectively (W. Crumb, personal communication), because these extreme values are assumed to be due to observational error. Accordingly, we truncate the Normal distribution in Equation (7) at 0 and 100, since these are imposed bounds on the data for % channel block (it would be better not to filter the data in this way, as, before making this adjustment, we observed repeated zero entries leading to the erroneous conclusion that there was almost no noise *σ* on the data).

**Figure 3.**
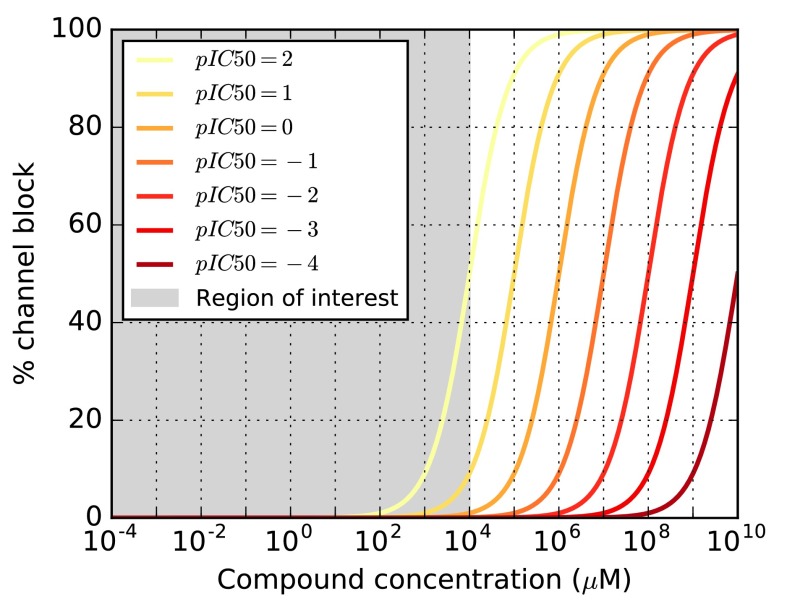
Large IC50 values are indistinguishable when they are orders of magnitude above the relevant concentration range. Here we show the effect of decreasing pIC50 (increasing IC50), while maintaining *Hill* = 1. The shaded “region of interest” covers the minimum and maximum concentrations in the data published by Crumb *et al.*. As pIC50 decreases, there is no significant change to the dose-response curve across the relevant range of concentrations — all predictions are close to zero response. As a result, we somewhat artificially ‘cap’ pIC50 priors to exclude pIC50 < −1, otherwise (for datasets with no response signal) convergence of minimisation and MCMC algorithms is difficult if not impossible.

Since *pIC*50, *Hill*, and *σ* are parameters that we infer, we need to specify a prior distribution across them, corresponding to *p*( ***θ***) in Equation (5). We choose independent uniform distributions for each parameter, but we could have chosen a more informative prior based on previous ion channel screening data, where available. We allow *Hill* to take values in (0,10). The Hill coefficient must be positive and describes the steepness of the dose-response curve, so after a certain point, increasing the Hill coefficient does not make a noticeable difference to the curve, so we choose 10 as a generous upper bound, above any biologically-plausible drug-binding we are aware of. Similarly, a compound that has no measurable effect could be thought of as having a very large IC50, and it makes no difference practically to model it as having an even larger IC50. This corresponds to a negative pIC50 value, and so we choose to allow *pIC*50 to take values in (-1,15) as values outside this interval will not have much effect (see Figure 3). We let *σ*, which is a standard deviation parameter and therefore also positive, take values in (0,50), where 50 is a generous upper bound for observational error, which we expect to be closer to 5–10% in practice.

As described in our previous publication ( Johnstone *et al.*, 2016a), we first perform a covariance matrix adaptation evolution strategy optimisation (CMA-ES, Hansen *et al.*, 2003) to find an optimal starting point for exploring possible parameter sets. We then use an adaptive Metropolis-Hastings MCMC algorithm ( Haario *et al.*, 2001) to infer *p*( *θ*| *data*), where *θ* = { *pIC*50, *Hill*, *σ*}. Briefly, we want to construct a sequence (Markov chain) of parameter-value sets that approximate samples from the left-hand-side of Equation (5). A set of parameter values is proposed by sampling from a multivariate Normal distribution centred on the most recent iteration’s parameter values. The posterior distribution ( Equation (6)) at these newly-proposed parameter values is then computed. The set of these proposed parameter values is accepted into the chain with a probability computed as the ratio of posterior distribution values between the current parameters and the proposed parameters. If the proposed set of parameter values is accepted, it is appended to the history of the chain and the next iteration will be taken from these new parameter values. As the MCMC algorithm runs, the covariance matrix of the proposal distribution skews in the directions where more sets of parameters are being accepted into the chain. After a large number of iterations, we discard the first quarter (or any suitably large fraction) of samples, known as ‘burn-in’ when the MCMC algorithm was still finding the best regions of parameter space. Then we plot normalised histograms of the remaining samples to approximate the posterior distribution.

The posterior distribution in the single-level model is given by (up to a factor of a constant)

$$p\left(pIC50,Hill,\sigma |\text{y}\right)\propto \prod_{j=1}^{K}p\left(y^{\left(i\right)}|pIC50,Hill,\sigma \right)p\left(pIC50\right)p\left(Hill\right)p\left(\sigma \right),\;(9)$$

where the first term on the right-hand-side is given by Equation (8).

### 3.2 Results

**Figure 4.**
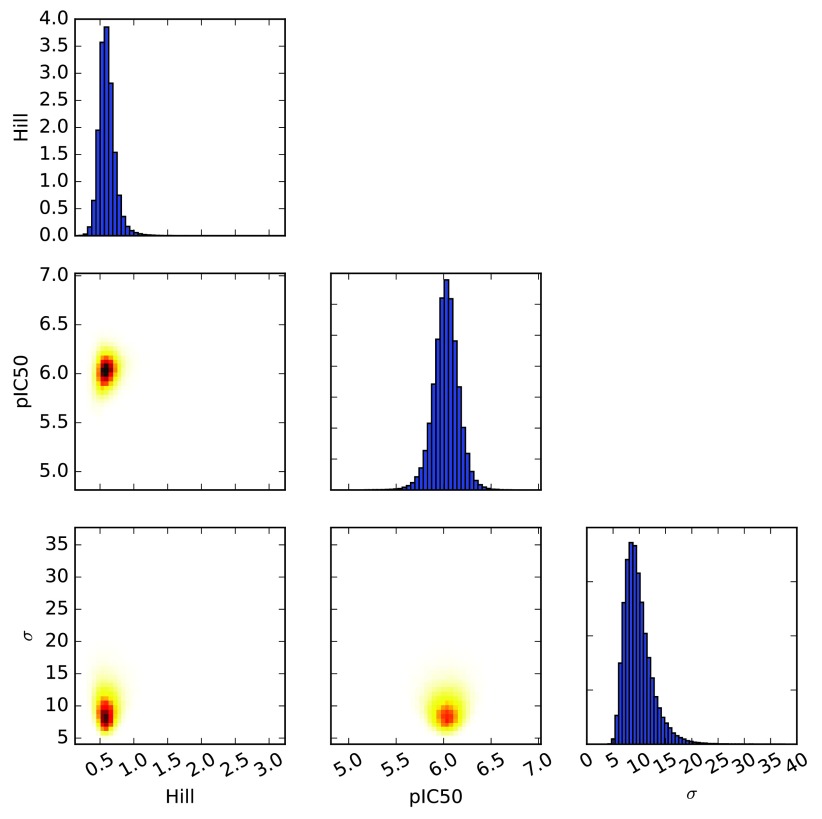
Matrix plot of normalised marginal and pairwise marginal histograms of the MCMC algorithm output samples for each parameter, in the amiodarone and hERG example. The well-defined narrow distributions, with lack of cross-correlation, suggest each parameter is being successfully inferred from the data.

In Figure 4 we plot normalised marginal and pairwise histograms for the values of the parameters for each sample of the MCMC algorithm output, which are approximate projections of the posterior distribution across these parameters. The spread in each distribution corresponds to the uncertainty in that parameter; if a parameter’s marginal posterior distribution is narrower, we are more certain about its value from the observed data.

**Figure 5.**
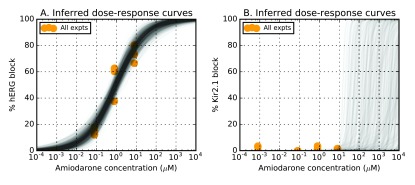
Inferred dose-response curves from MCMC ( *pIC*50, *Hill*) samples. **A**: amiodarone with measurable effect on hERG. **B**: amiodarone with no measurable effect on Kir2.1. Outside of the range of measured concentrations, the MCMC algorithm was unable to find narrow ranges for possible parameter values, because the experimental data does not contain enough information.

Before propagating these uncertainties, we first draw ( *pIC*50, *Hill*) samples from the MCMC output, and plot dose-response curves with these parameter values. Examples are given in Figure 5, where amiodarone has a measurable blocking effect on hERG, but no measurable effect on Kir2.1. As we take more samples, the plotted curves build up a distribution of possible dose-response curves given the experimental data. For each compound concentration, we then have a range of possible responses with their relative probability densities being given by the density of dose-response curves at that concentration.

## 4 Hierarchical (multi-level/mixture) model

**Figure 6.**
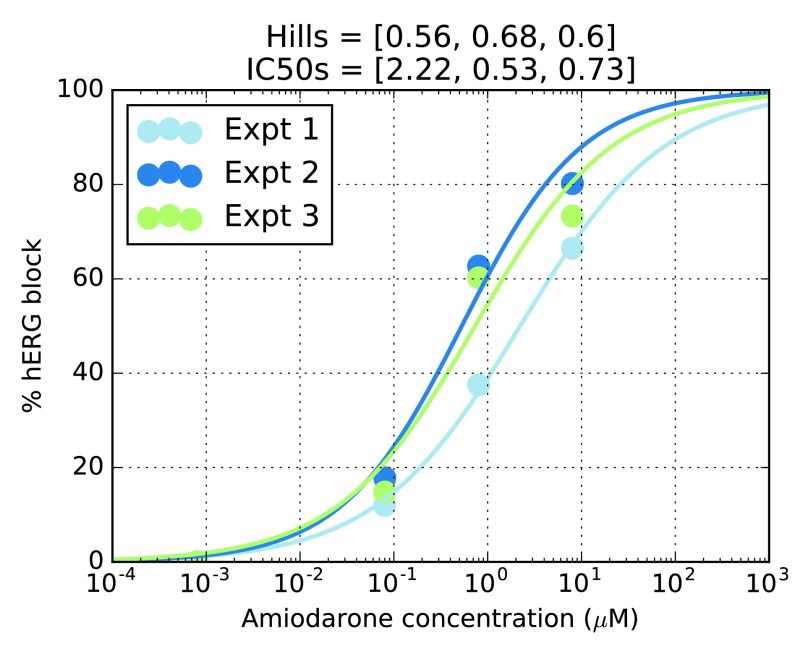
Data for hERG block by amiodarone suggests inter-experiment variability. Different whole-cell patch-clamp experiments are plotted with different colours. In this case, the responses are consistently in the same ordering relative to the other experiments at each compound concentration ( *e.g.* Experiment 2 always shows the largest response, and Experiment 1 always the smallest). This suggests inter-experiment variability that is distinct from observational error *σ*.

When we plot the ion channel screening data and group the data points according to their respective experimental repeats, instead of treating them as data points from one experiment as in Section 3, we see that data points from the same experiment generally keep their relative position. That is, we often see that the highest value at each concentration was observed during the same experiment, as shown for the amiodarone and hERG case in Figure 6.

### 4.1 Methods

The intra-experiment correlation seen in Figure 6 suggests that each experiment has its own distinct properties. We model this as inter-experiment variability in the pIC50 value and Hill coefficient. That is, we treat each experiment as having its own *p*IC *50* and *Hill*, which are drawn from distributions which are shared across experiments. We are assuming that two experiments have different pIC50 values, say, but that these values are mutually informative. We let *N _e_* be the number of experiments performed. The vector of data points obtained from experiment *i* is **y _i_**, where *i* = 1, …, *N _e_*.

We take a hierarchical approach and assume that there is some ‘higher-level’ distribution that governs how these parameters vary across experiments (see Congdon, 2010, for an introduction to this approach).

**Figure 7.**
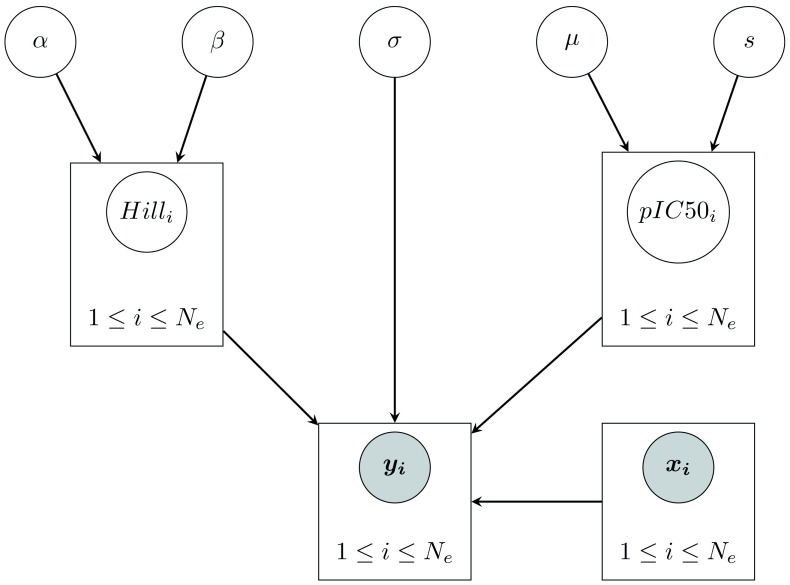
Hierarchical statistical model for dose-response data. *i* indexes the individual experiments. All non-shaded variables are parameters for which we wish to infer probability distributions.

Hill coefficient and pIC50 distributions for ion channel screening datasets of up to *N _e_* > 12,000 repeats were published in Elkins *et al.* (2013), there they were found to fit independent log-logistic and logistic distributions, respectively. We assume the same type of distributions would occur here (if the experiments were repeated enough): that is, each experiment’s *Hill _i_* is drawn from a log-logistic distribution with parameters *α* and *β*; and each experiment’s *pIC*50 _*i*_ is drawn from a logistic distribution with parameters *μ* and *s*. We have assumed that the observational errors are drawn from the same Normal distribution across all *N _e_* repeats, so we infer just a single noise standard deviation parameter *σ*. A schematic of this hierarchical statistical model is given in Figure 7, with the ‘mid-level’ parameters and ‘bottom-level’ data points being independently distributed according to

$$Hill_{i}∼\text{log}-\text{logistic}\left(\alpha ,\beta \right),\;(10)$$

$$pIC50_{i}∼\text{logistic}\left(\mu ,s\right),\;(11)$$

$$y_{i}^{\left(j\right)}∼\left(f\left(x_{i}^{\left(j\right)};pIC50_{i},Hill_{i}\right),\sigma^{2}\right),\;(12)$$

where $y_{i}^{\left(j\right)}$ is the *j* ^th^ concentration entry in experiment *i*’s responses **y**_i_. We suppose that every experiment *i* has *K _i_* data points (to generalise to cases where different experiments tests different numbers of concentrations), so *j* = 1, …, *K _i_* and *i* = 1, …, *N _e_*.

**Figure 8.**
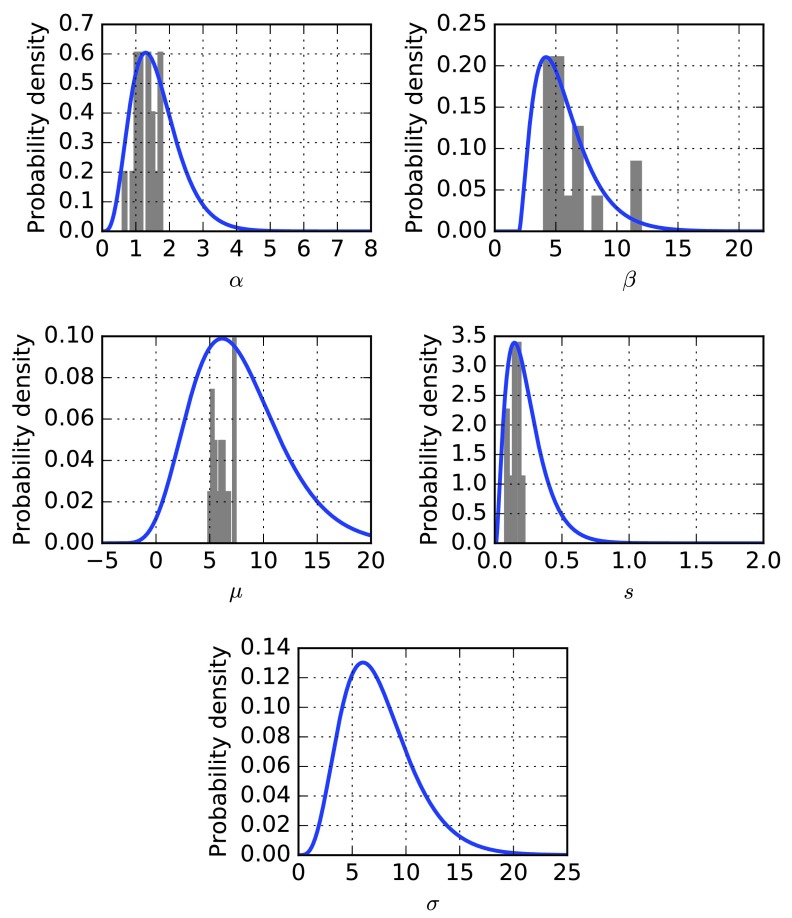
Prior distributions. Blue: gamma distributions used as the prior distributions over *α*, *β*, *μ*, *s* and *σ*. Grey: histograms of parameter estimates for different strongly-blocking control compounds, with large numbers of repeats, as previously published in Elkins *et al.* (2013). However, we want to be able to fit to all compounds, including ineffective ones that elicit no response, hence the increased width of the prior distributions on certain parameters ( *μ* particularly).

We now need to specify prior distributions over the ‘top-level’ parameters (in Figure 7): *α*, *β*, *σ*, *μ*, and *s*. Prior distributions are chosen to contain any prior information or beliefs we have about the parameters before observing the data. We can therefore inform our choice of prior distributions by considering previously-published ion channel screening data. We use gamma distributions for all of these, since gamma distributions, in general, only put probability mass on positive values, and because these parameters are all positive, with the exception of *μ*. *μ*, however, can take any value and represents the centre of the logistic distribution. For *μ*, we used a gamma distribution which is shifted along the *x*-axis down to -4, so there is little probability mass below -2. We choose this because as the pIC50 value becomes lower and lower, the IC50 becomes larger, and eventually any possible compound effects occur well above the experimental concentrations. This was illustrated above in Figure 3, where dose-response curves (with *Hill* = 1) have been plotted for varying values of *pIC*50. The shaded “region of interest” covers the minimum and maximum concentrations in the data published by Crumb *et al.* Ion channel screening is generally performed at concentrations that range to well-above therapeutic concentrations, and so we do not want to infer how a compound will behave at even higher concentrations. These gamma prior distributions were tuned to cover values provided by Elkins *et al.* (2013), but also allow more room for variation. Plots of the prior distributions for *α*, *β*, *μ*, *s*, and *σ* are given in Figure 8.

In addition to covering the values published by Elkins *et al.*, we restricted *β* to be greater than 2, so that all log-logistic distributions generated would have no probability mass at 0, and also the gradient of the probability density function would be zero. This prevents Hill coefficients equal to 0 from being sampled by the MCMC algorithm. We also enforce that *σ* be greater than 10 ^−3^, since we believe there is always the possibility of observation error, and hence there must be a positive standard deviation, we also run into division-by-zero numerical problems with the evaluation of the target distribution if we sample *σ* = 0 (see Equation (8)).

**Figure 9.**
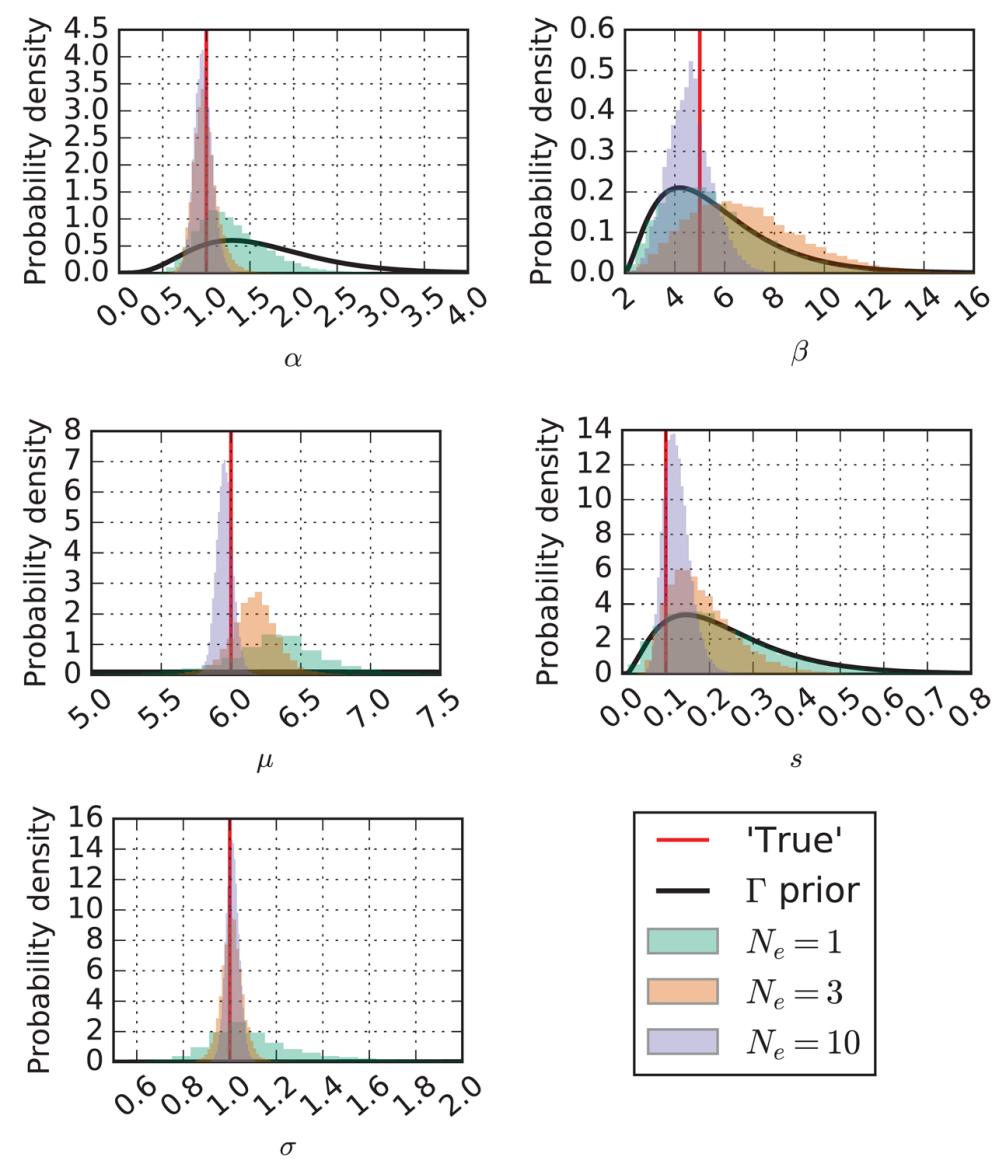
A comparison between marginal posterior distributions for ‘top-level’ parameters in the hierarchical model, with their respective gamma (Γ) prior distributions. The number of (synthetic) experimental datasets, *N _e_*, being fitted was increased. As we fit to more experiments, the prior distributions have a smaller effect on the posterior distributions. Where the black line for the prior distributions looks as if it lies along the *x*-axis (for *μ* and *σ*, the prior distribution was much wider than the marginal posterior distribution; there is a lot of information on these parameters with just one experiment).

The choice of prior distribution will have an effect on the posterior distributions (via Equation (6)). However, the more information that is contained in our data, the less effect we expect our prior distribution to have on our posterior distribution. For example, Figure 9 shows how the marginal posterior distribution for the ‘top-level’ parameters correspond to their respective prior distributions in the case of synthetic data, where we fit to different numbers of experimental datasets. This synthetic data were generated by sampling pIC50 values from a logistic distribution with *μ* = 6 and *s* = 0.1, and Hill coefficients were sampled from a log-logistic distribution with *α* = 1 and *β* = 5. Normally-distributed observation noise with standard deviation *σ* = 1 was added to the dose-response model at every concentration.

We want to infer the posterior probability distribution for *α*, *β*, *μ*, *s*, *σ*, *Hill _i_* and *pIC*50 _*i*_, for *i* = 1, …, *N _e_*, giving a total of 5 + 2 *N _e_* parameters. Using Bayes’ Theorem, the posterior distribution in Equation (6) is now given by

$$\begin{array}{l} p\left(\alpha ,\beta ,\mu ,s,\sigma ,{\left\{Hill_{i}\right\}}_{i=1}^{N_{e}},{\left\{pIC50_{i}\right\}}_{i=1}^{N_{e}}|{\left\{{\text{y}}_{\text{i}}\right\}}_{i=1}^{N_{e}}\right)\\ \;\propto \left(\prod_{i=1}^{N_{e}}p\left({\text{y}}_{\text{i}}|\sigma ,Hill_{i},pIC50_{i}\right)p\left(Hill_{i}|\alpha ,\beta \right)p\left(pIC50_{i}|\mu ,s\right)\right)p\left(\alpha \right)p\left(\beta \right)p\left(\mu \right)p\left(s\right)p\left(\sigma \right). \end{array}\;(13)$$

We use the same adaptive Metropolis-Hastings MCMC algorithm as in Section 3.1 to infer a posterior distribution from the experimental data. Since we have many more parameters than in the non-hierarchical case, we expect to have to run our MCMC algorithm for more iterations to adequately approximate the posterior distribution. For most cases in this dataset, we have *N _e_* = 3 or *N _e_* = 4, which is not too demanding since our mathematical model is a simple analytic expression, and does not require solving differential equations as our previous work did ( Johnstone *et al.*, 2016a). However, if *N _e_* became very large, we may have to use alternative MCMC techniques.

### 4.2 Implementation

Our tool *PyHillFit* ( Johnstone *et al.*, 2016b) takes a CSV file of dose-response points as its input. The file should be comma separated values (.CSV) in the following format for each line:

compound name, channel name, experiment number, dose (μM), response (% inhibition)

The Monte Carlo algorithms were written in Python using **NumPy** 1.11.0 for numerical linear algebra ( van der Walt *et al.*, 2011), and functions from the **SciPy** 0.15.1 library ( Jones *et al.*, 2001). **Pandas** 0.17.1 was used to read the input data csv files ( McKinney, 2010). **cma** 1.1.6 was used for initial optimisation to find best-fit parameter values ( Hansen *et al.*, 2003), which act as starting positions for the MCMC algorithms. All figures were plotted in **matplotlib** 1.5.1 ( Hunter, 2007) and **seaborn 0.7.1** ( http://seaborn.pydata.org/).

PyHillFit output takes the form of files listing samples of the posterior distributions for the dose-response curve parameters, together with some visualizations of these (as shown throughout this article).

### 4.3 Results

**Figure 10.**
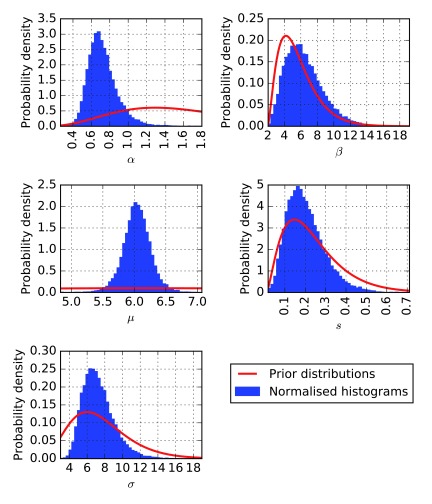
Normalised marginal histograms for the ‘top-level’ parameters, *α*, *β*, *μ*, *s*, and *σ* after running the fitting the hierarchical model to the amiodarone and hERG dataset using the MCMC algorithm. The red lines indicate the respective prior distributions. Most of these distributions are narrower than their respective prior distributions in Figure 8, with the exception of *β*. We therefore conclude that the experimental data does not contain much information about *β*, in line with the synthetic data study shown in Figure 9.

As before, we plot normalised marginal histograms to approximate the marginal posterior distributions for each parameter. Such histograms are plotted for *α*, *β*, *μ*, *s*, and *σ* in Figure 10 for the amiodarone and hERG case. We can compare these to the prior distributions shown in Figure 8, and we see that in most cases we have much narrower marginal posterior distributions than prior distributions. This tells us that the data contains enough information about those parameters to constrain them to narrower intervals.

**Figure 11.**
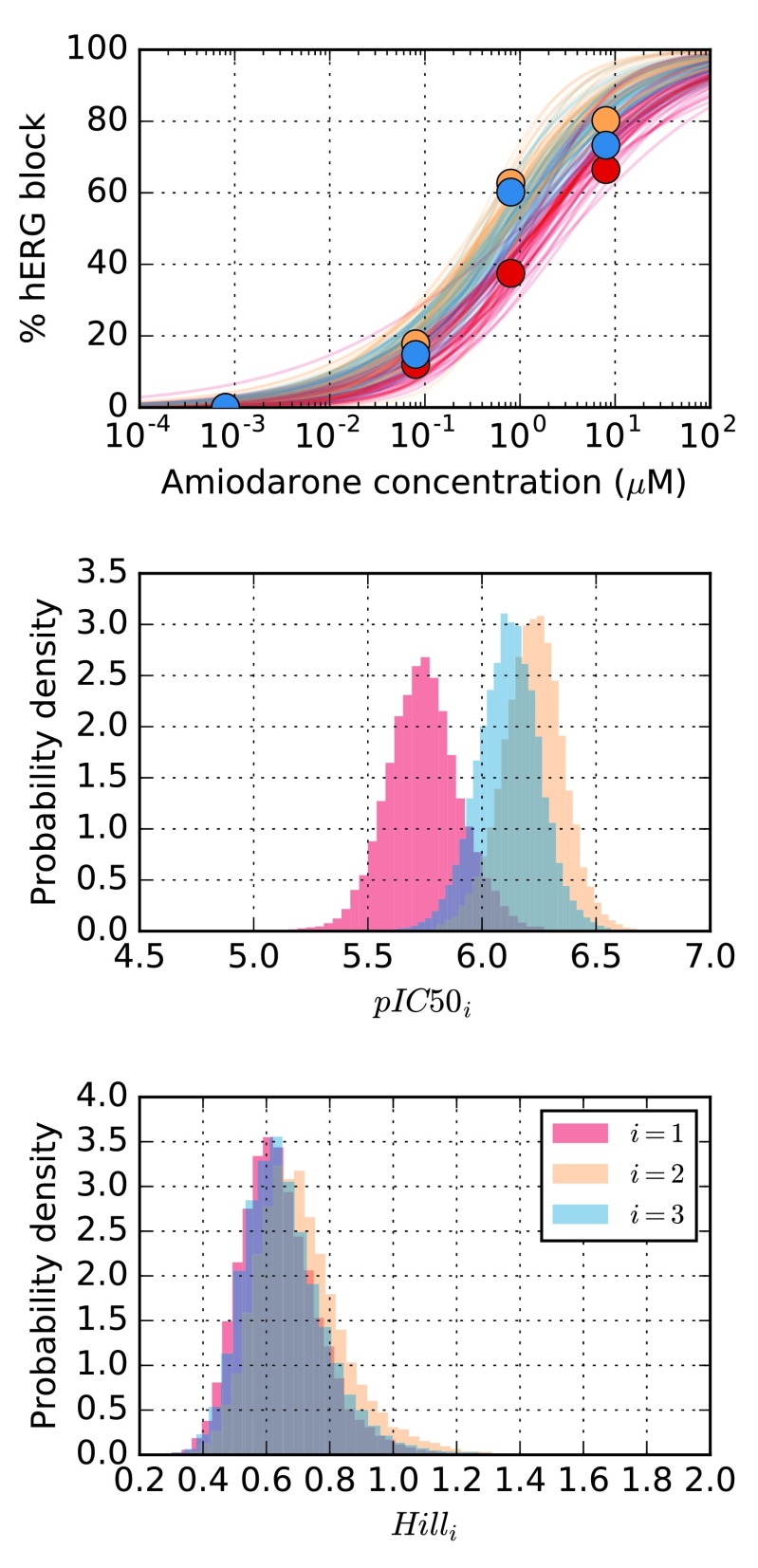
Inferred parameters for individual experiments. Top: dose-response curves plotted using the ‘mid-level’ *pIC*50 _*i*_ and *Hill _i_* samples from our MCMC algorithm output from the amiodarone and hERG dataset. Middle & bottom: superimposed normalised histograms for *pIC*50 _*i*_ and *Hill _i_*, after fitting our hierarchical model to the amiodarone and hERG dataset using the MCMC algorithm. We find that the Hill coefficient does not vary much between experiments, however there is variability within the pIC50 value.

We can also superimpose the normalised histograms for each *Hill _i_* and *pIC*50 _*i*_ to give us an idea of how much inter-experiment variability is present in these parameters. These superimposed histograms are plotted in Figure 11 for the amiodarone and hERG case.

To make predictions about how a particular compound and channel will interact if we perform another experiment, we consider the *posterior predictive* distributions for *Hill _i_* and *pIC*50 _*i*_. That is, what are *p*( *Hill*_*N*_e+1__|data) and *p*( *pIC50*_*N*_e+1__|data)? Since the *Hill _i_* and *pIC*50 _*i*_ are modelled as being drawn from log-logistic and logistic distributions, respectively, we sum the log-logistic and logistic distributions generated from the ‘top-level’ parameters at every iteration of our MCMC algorithm output, then normalise them to obtain two new probability distributions.

$$p\left(Hill_{N_{e}+1}|\text{data}\right)\approx \frac{1}{T}{\text{Σ}}_{t=1}^{T}\text{log}-\text{logistic}\left(Hill_{t};\alpha_{t},\beta_{t}\right),\;(14)$$

$$p\left(pIC50_{N_{e}+1}|\text{data}\right)\approx \frac{1}{T}{\text{Σ}}_{t=1}^{T}-\text{logistic}\left(pIC50_{t};\mu_{t},s_{t}\right),\;(15)$$

where *t* indexes the samples in our Markov chain, after having discarded a number of initial samples as a burn-in.

**Figure 12.**
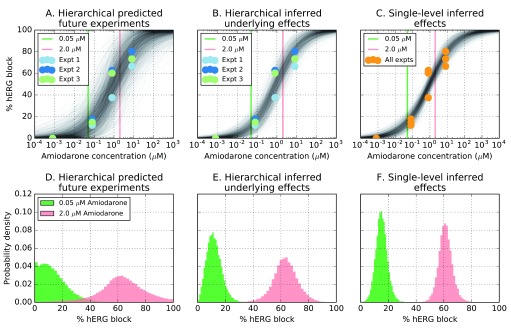
Predicted dose-response curves and associated probability distributions for levels of block at example concentrations when fitting to the amiodarone and hERG dataset. **A**: Hierarchical model — predictions for how a future experiment will behave, with samples taken from the posterior predictive distributions. **B**: Hierarchical model — inferred distribution for the underlying behaviour of the system, plotted by using *μ* and *α* samples from the MCMC algorithm output directly as values for *pIC*50 and *Hill*, respectively. **C**: Single-level — inferred distribution, plotted by taking samples from the MCMC algorithm output. **D**, **E**, **F**: Histograms of the intersections between the vertical lines and dose-response curves in **A**, **B**, **C**, respectively, at two different concentrations of amiodarone.

These are not necessarily distributions which can be sampled from directly, but we can approximately sample from them using the inverse-cumulative distribution function (CDF) method. We sum and then normalise the individual log-logistic and logistic CDFs. After sampling from these new distributions, we plot similar dose-response curves as in Section 3.2. A plot of predicted dose-response curves for a future experiment, following the hierarchical model MCMC, is given in plot A of Figure 12. To make a prediction of what %-block will be induced by that compound at a particular concentration, we take a vertical cross-section through these dose-response curves and plot a normalised histogram of these levels of block to approximate a probability distribution, as shown in plot B of Figure 12.

Note that the hierarchical model allows us to make two sets of predictions. Firstly, using the posterior predictive distribution given by Equation (14) & Equation (15) as shown in Figure 12 (panel A). This distribution includes inter-experiment variability, and can therefore be considered a distribution that predicts where data points from future experiments may lie. Secondly, we can examine the variability in the underlying properties of the compound; the ‘average’ effect, before it is altered by inter-experiment variability (panel B). We generated this plot by taking samples of *α* and *μ* to use as Hill and pIC50 values. We would expect panel B to be more directly comparable with the single-level approach (which fits ‘average’ data points), which is shown in panel C for comparison.

Which of the two distributions (illustrated in panels A or B in Figure 12) one may wish to use for predictions is subtle. If we consider that the source of variability between experiments is also present in the system that we are making predictions for, then the first case (panel A) would be the best to use. If however we consider that there is a single underlying effect, and the act of measuring it introduced inter-experiment variability that is not present in the real system, then the second distribution (panel B) would be more appropriate. Most biological experiments implicitly assume the second case is true — that by taking repeated measurements and then taking the average, a more accurate assessment of the underlying system is made.

## 5 A comparison of single-level and hierarchical models

There are advantages and disadvantages to choosing either the single-level statistical model, or the hierarchical statistical model. The main benefit of the single-level model is that we are only fitting three parameters, meaning that the parameter space of interest is relatively easy to explore. This means that we need to run our MCMC algorithm for fewer iterations to obtain an acceptable approximation of the posterior distribution than if we had a larger number of parameters, reducing overall computation time. However, a model with an analytic solution such as the one we have here ( Equation (3)) can be solved very quickly, and so computation time is generally not a problem for even the hierarchical version of the model, for this application. There is also little sensitivity to the prior distributions, as there is a lot of information about all three parameters in even one experiment.

In Figure 12 we can compare the results of the two types of inference for real data on amiodarone block of the hERG current. There is a small difference in the predicted curves.

In the single-level case, by fitting to all data points at once, the inference can misinterpret inter-experiment variability and assign it to the ‘wrong’ parameter(s). To demonstrate this, we generated two sets of synthetic data corresponding to these fictitious compounds, with fictitious inter-experiment variability properties:

1. For shamiodarone, *Hill* was fixed as 1 across all experiments, and the *pIC*50 _*i*_ were drawn from a logistic distribution, with *μ* = 6 and *s* = 0.2.
2. For shamitriptyline, *pIC*50 was fixed as 6 across all experiments, and the *Hill _i_* were drawn from a log-logistic distribution with *α* = 1 and *β* = 2.5.

In both cases, we simulated 5 experiments where each experiment consists of measuring % channel block at 4 different compound concentrations. We added Normal observation noise with standard deviation *σ* = 0.5 to each point.

### Case 1: fixed Hill and varying pIC50

**Figure 13.**
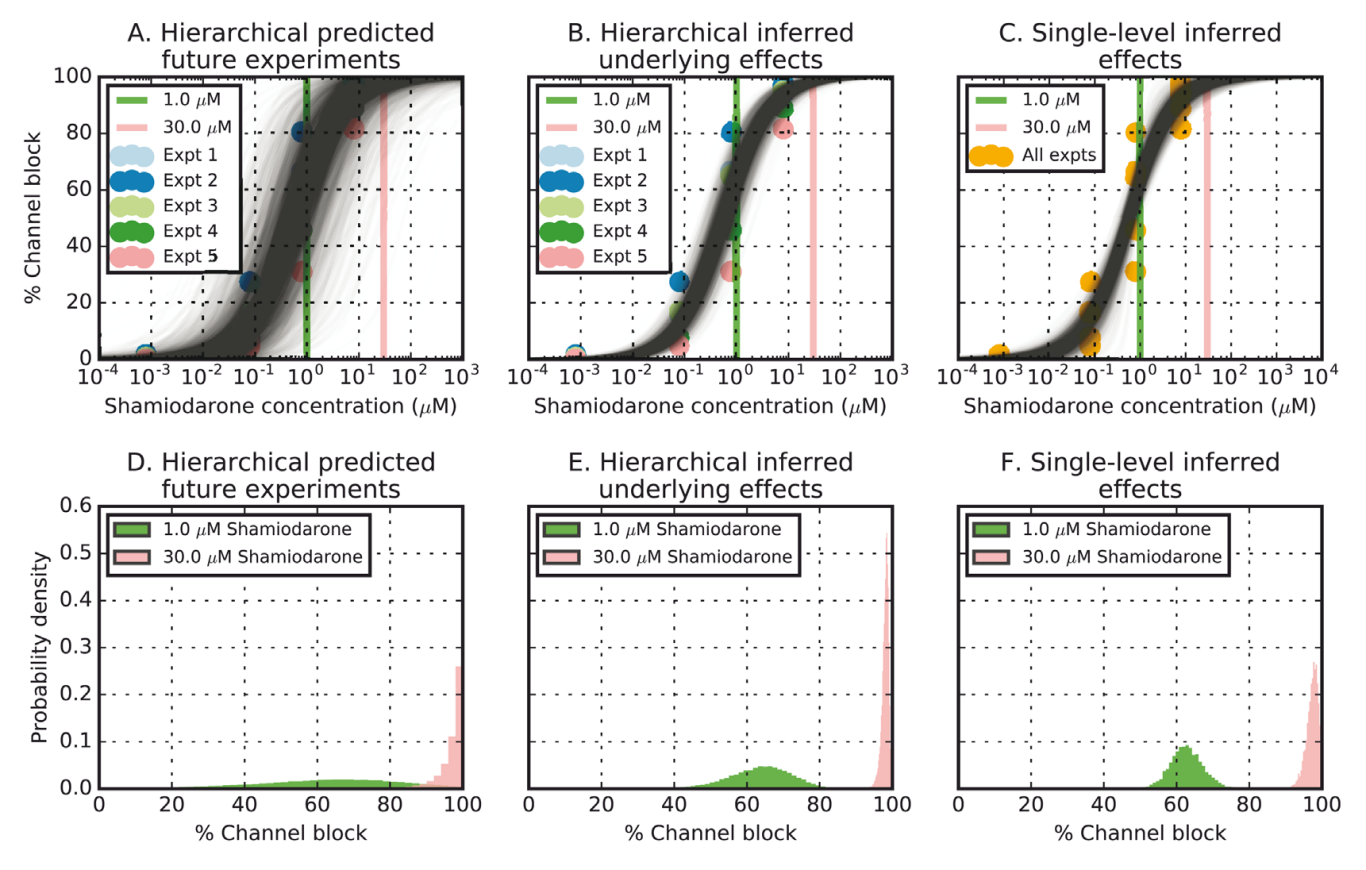
Inference on synthetic data generated by fixing *Hill* = 1 and varying *pIC*50. **A**: Predicted dose-response curves, with *pIC*50 and *Hill* sampled from their respective posterior predictive distributions, taking inter-experiment variability into account. **B**: Inferred underlying behaviour of the compound-ion channel interaction, with inter-experiment variability discounted. **C**: Inferred dose-response curves from single-level inference. **D**– **F**: Normalised histograms of the cross sections plotted with vertical lines in plots **A**– **C**. These histograms represent probability density functions of % block at a particular concentration, given the (synthetic) experimental data.

A sample of the inferred curves for this case are given in Figure 13. The plots in panels A & D represent *pIC*50 and *Hill* parameter values being drawn from their respective posterior predictive distributions; this gives predictions of how we believe the observations from a future experiment would behave. The plots in B & E are based on *α* and *μ* samples — what we believe to be the underlying ‘average’ behaviour of the compound interacting with an ion channel, when experimentally-introduced variability is discounted. The hierarchical model was able to identify consistency within the *Hill _i_*, and the MCMC algorithm generally only infers that *pIC*50 _*i*_ varied between experiments.

The histograms in Figure 13 (panels D–F) are a cross-section of the dose-response curves at different concentrations, and represent the probability density of % block at that compound concentration. Note that each curve in panel B has approximately the same slope, corresponding to a consistent Hill coefficient, whereas in panel C we see that there is a greater range of slopes, corresponding to (slightly more) variability in the Hill coefficient. Comparing plot E with plot F, we see that at 0.05 *μ*M concentration, the non-hierarchical model is less certain about the % channel block than the hierarchical model, because the former has incorrectly inferred there is more variation in the Hill coefficient. So the single-level model tends to compensate for the varying parameter values by fitting curves that fit through an ‘average’ of the points. The algorithm does this by varying both *Hill* and *pIC*50 to obtain curves that could fit the data reasonably well, even when the synthetic data were generated by holding one parameter fixed and varying the other.

### Case 2: fixed pIC50 and varying Hill

**Figure 14.**
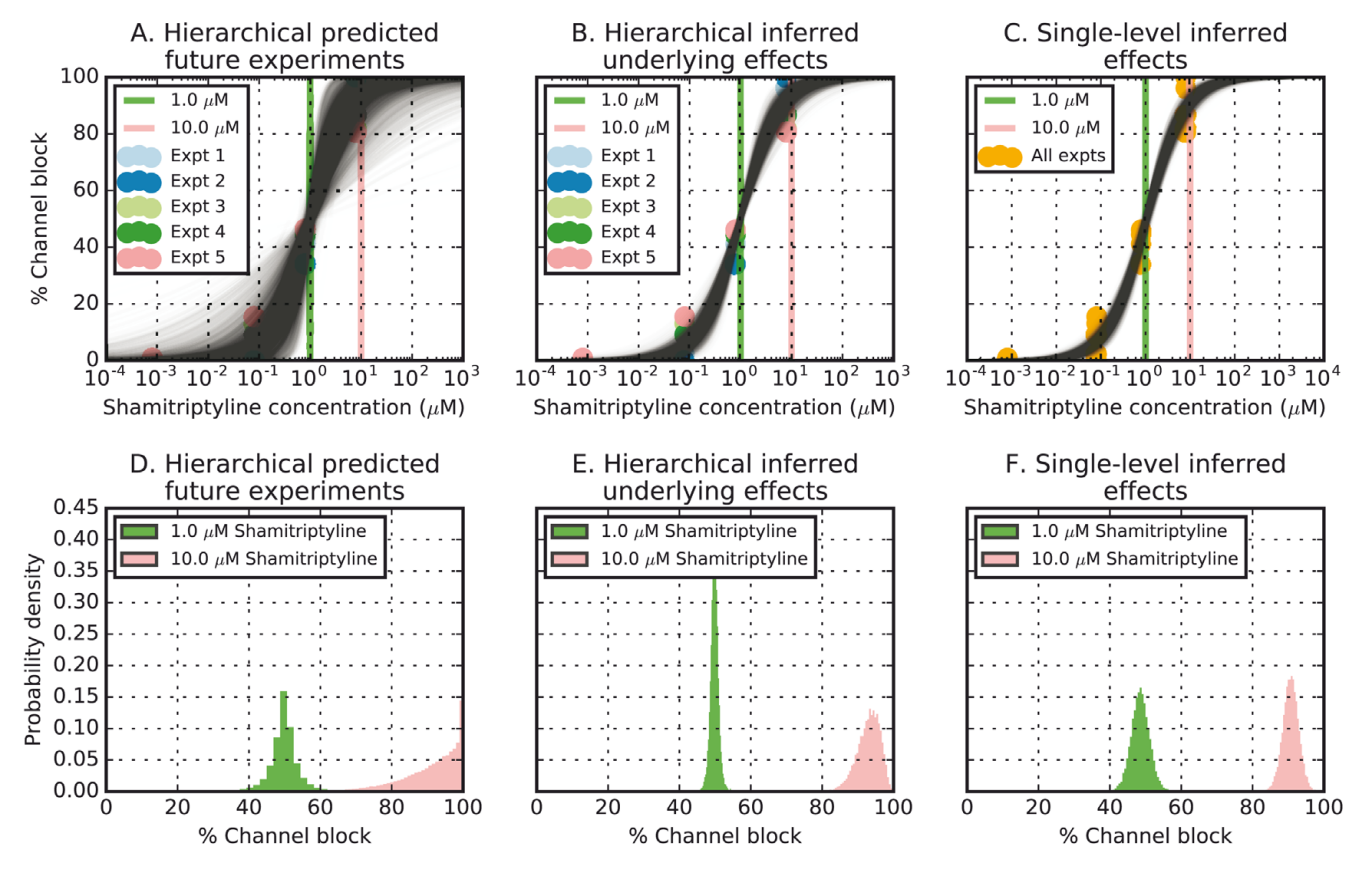
Inference on synthetic data generated by fixing *pIC*50 and varying *Hill*. **A**: Predicted dose-response curves, with *pIC*50 and *Hill* sampled from their respective posterior predictive distributions, taking inter-experiment variability into account. **B**: Inferred underlying behaviour of the compound-ion channel interaction, with inter-experiment variability discounted. **C**: Inferred dose-response curves from single-level inference. **D**– **F**: Normalised histograms of the cross sections plotted with vertical lines in plots **A**– **C**. These histograms represent probability density functions of % block at a particular concentration, given the (synthetic) experimental data.

A sample of the inferred curves for this case are given in Figure 14. The single-level model in panel C does show small variability in the Hill coefficient, as well as small variability in the IC50 (and hence pIC50). This leads to a reasonably spread prediction of ion channel block at both a concentration near pIC50 and at a higher concentration (panel F). But we know that the underlying data had the same pIC50, and so variability near the IC50 should be minimal, and indeed the spread of predictions at a higher concentration should be larger. The hierarchical model captures the desired underlying variability better (compare panels E and F). The Hill coefficient varies (panel B) while also capturing the low variability in the pIC50 value (there is still some variability in the inferred pIC50 distribution due to observational noise and a low number of repeat experiments). As a result, the predicted percentage blocks in panels E and F are different. As for Case 1, either too-much or too-little variability is predicted by the single-level inference, depending on the concentration.

## 6 Propagating dose-response uncertainty

The proposed Comprehensive *in-vitro* Pro-arrhythmia Assay (CiPA) recommends the use of computational action potential models in the drug safety process. Ion channel screening will be performed, and the IC50 values and Hill coefficients obtained from these experiments are to be used in action potential models to predict whether or not a compound is likely to be pro-arrhythmic. One simple proposed measure of pro-arrhythmia is action potential duration prolongation ( Mirams *et al.*, 2011), directly related to prolongation of the QT-interval, which can be a precursor to potentially fatal arrhythmias such as Torsade de Pointes.

Using best-fit IC50 values and Hill coefficients obtained from ion channel screening data, we can compute a predicted level of block of each of the ion currents in an action potential model, at a particular compound concentration. We then simulate an action potential and measure the action potential duration prolongation relative to the control case (see Beattie *et al.* (2013) for an example of this approach). However, when using best-fit IC50 values and Hill coefficients, we obtain a single predicted action potential after simulating a particular compound concentration.

Instead, we use our tool to infer probability distributions for each experiment’s IC50 value and Hill coefficient. Each sample is ‘equally likely’, but there will be more samples around the regions of greater probability density. We can then randomly take samples from these distributions and compute an action potential duration for each sample. Again, each output is ‘equally likely’, but there will be more outputs close to each other where there is the greatest probability density. This allows us to construct a probability distribution for predicted action potential durations based on the original ion channel screening data (uncertainty propagation).

**Figure 15.**
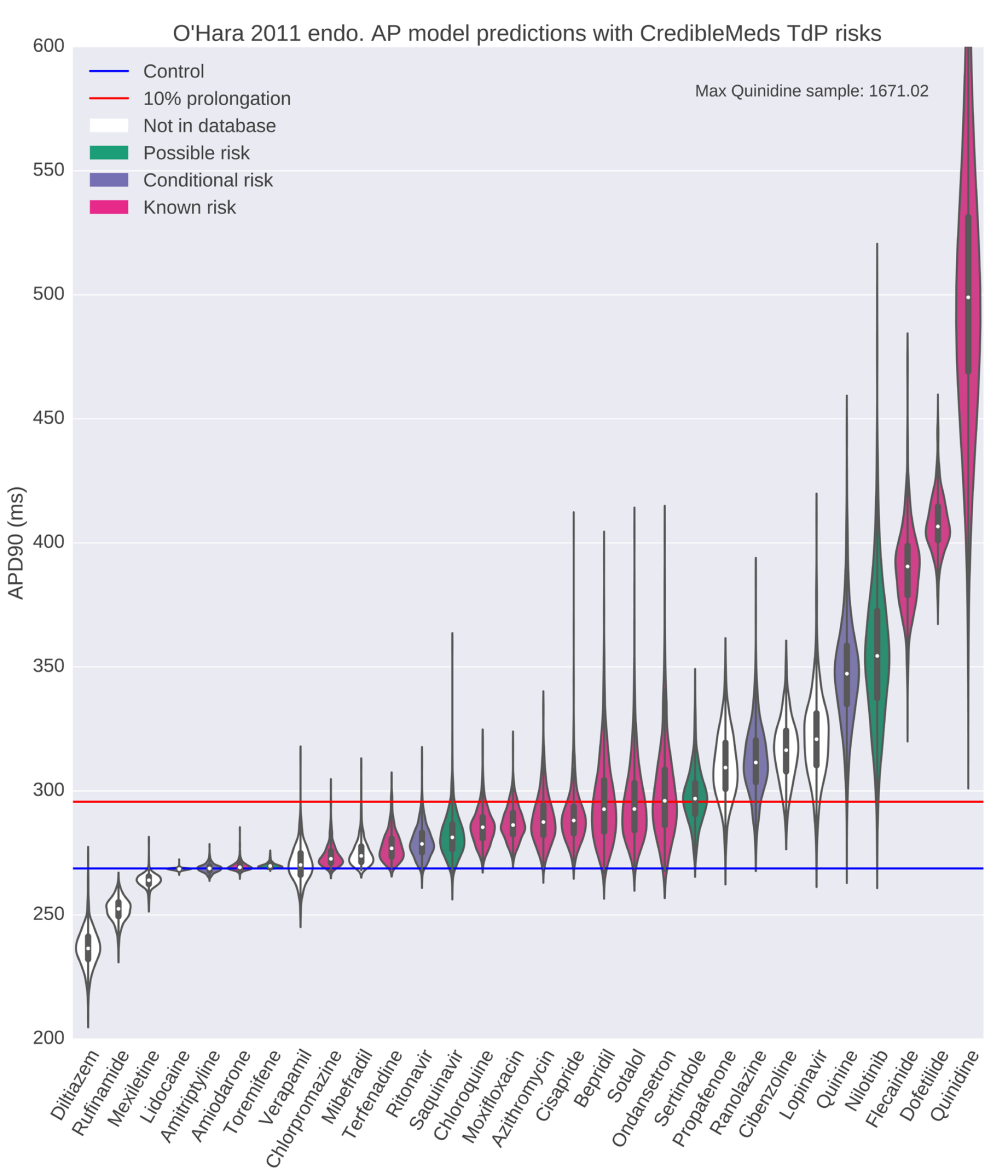
Violin plots of prediction action potential duration (APD90, time taken for the cell to return to 90% repolarisation after depolarisation) using 500 samples from the iterations of our MCMC algorithm under the hierarchical statistical model. Simulations were run using the O'Hara *et al.* (2011) human ventricular cardiomyocyte action potential model, and APD90s were computed. We used ‘AP-predict’ ( Williams & Mirams, 2015), a bolt-on project for the Chaste open-source computational biology C++ library ( Mirams *et al.*, 2013). Violin distribution plots are shown for each of the 30 compounds discussed by Crumb *et al.* In one case, several samples led to very long action potentials and so the y-axis is cut off early for clarity.

To illustrate the proposed uncertainty propagation we use our tool to fit hierarchical dose-response parameters to thirty drug compounds for seven ion currents each using the Crumb *et al.* (2016) dataset supplied with the code associated with this article. We then take 500 samples from the MCMC output for each drug and channel combination, based on the 'underlying effects' curves from hierarchical fits (see Figure 12, Figure 13 & Figure 14), and simulate action potential durations after applying seven ion channel block using the approach outlined in Mirams *et al.* (2011). We plot the resulting predicted action potential duration distributions as violin plots in Figure 15. A violin plot for each of the thirty compounds is coloured according to the Torsade/QT risk categorisation in the CredibleMeds database ( https://crediblemeds.org/). We also plot the control action potential duration in the O'Hara model along with a 10% action potential duration prolongation.

We see that the vast majority of the compounds have overlapping probability distributions for predicted APD at maximum free therapeutic plasma concentration, suggesting that at least this previously-proposed measure will be insufficient to distinguish compounds in terms of risk based on data such as these.

This suggests that either: action potential prolongation is not a good enough marker of pro-arrhythmia; or, there was too much uncertainty associated with the experimental data to constrain these distributions to narrow distinct ranges. To counter the latter point, we suggest that more experimental repeats be performed. In either case, it is imperative to realise that the data being used have a level of uncertainty which means it is not possible to rank the majority of these drugs in terms of their predicted APD.

## 7 Discussion

A Bayesian framework is a useful tool to address uncertainty characterisation in ion channel screening data. When no uncertainty characterisation is performed, one can obtain best-fit parameter values for the data presented, but there is no associated probability in terms of the behaviour that generated the data, or in predictions informed by the data. The single-level Bayesian inference model can provide ranges of possible dose-response curves (and underlying parameters) that fit ion channel screening data. But parameter-specific inter-experiment variability can be missed when using a ‘single-level’ statistical model, as the algorithm treats all points equally and so varies the parameters without considering the inter-experiment correlations. This leads to an ‘averaging’ effect, where the dose-response model is fitting to an average of the experimental data points, but may not reflect the behaviour of any individual experiment. However, this single-level inference is quick to run as it only requires fitting 3 parameters, and provides a better approximation of probability distributions than a single best-fit.

A hierarchical statistical model can capture inter-experiment variability within certain dose-response parameters, as demonstrated in the synthetic cases discussed in Section 5. The hierarchical model can therefore be used to infer inter-experiment behaviour, and hence predict how a future experiment might behave. By taking samples for the ‘top-level’ parameters from our MCMC output, we can build distributions of how we believe the compound is interacting with the ion channel. At a given compound concentration, we then have a probability distribution for possible levels of ion channel block. The hierarchical model is able to determine what variability is being introduced at the experimental level, and allows us to make probabilistic statements about the underlying behaviour.

Our hierarchical model is similar to *nonlinear mixed effects* (NLME) modelling, but we operate in a Bayesian framework. NLME assumes a similar structure to that shown in Figure 7, but infers best-fit values for the ‘top-level’ parameters, and a distribution from which the ‘mid-level’ parameters are sampled. While it does capture inter-experiment variability and would allow us to make predictions about how a future experiment might behave, it only provides a point-estimate for underlying behaviour, rather than different possibilities with relative probabilities.

A possible limitation of the hierarchical model is that computation time increases with the number of experimental datasets being fit to at once. This is not a problem for up to 4 or 5 experiments, but the number of parameters quickly becomes intractable for the adaptive-Metropolis MCMC algorithm that we have been using. In general, with MCMC methods, we want to run our algorithm for as long as possible, to best approximate samples from the posterior distribution. There is therefore no upper limit for how long this method takes, although for these examples we have run our algorithm for 500,000 iterations, which takes approximately 12 minutes for the amiodarone-hERG case which has 3 experimental datasets of 4 concentrations each.

Another possible limitation of the hierarchical model is the dependence on the prior distributions for the ‘top-level’ parameters. As shown in Figure 9, when there is not much data, the posterior is heavily influenced by the prior. However, we chose our priors based on data published by Elkins *et al.* which was based on 12,000 ion channel screening experiments, and we therefore have some confidence in their shapes ( Figure 8). In a Bayesian framework, should new data become available, we can compute new posterior distributions for the parameters according to Equation (5), by using a previous posterior distribution as the new prior distribution.

A benefit of both inference techniques is that we introduce the observation noise as a parameter to be fitted, along with the pIC50 values and Hill coefficients. Instead of estimating the observation noise and then fitting dose-response curves based on our estimate, we allow the MCMC algorithm to find likely levels of noise, while also quantifying the uncertainty in those estimates. Since all of these parameters are being fit at the same time by the MCMC algorithm, we can extract how much noise on dose-response parameters is introduced by inter-experiment variability, and how much noise is due to observation error.

### 7.1 Conclusions

Single best-fit parameter estimates from ion channel screening data can give a *most likely* set of dose-response curve parameters. However, this approach does not provide us with a measure of uncertainty around these parameters. The software tool we present can quantify some of the uncertainty associated with dose-response curves, with its default priors set for ion channel screening data, for the purposes of propagating this uncertainty into further quantitative studies.

## Data and software availability

Latest source code and datasets used in the publication: https://github.com/mirams/PyHillFit

Archived source code and datasets as at the time of publication: https://doi.org/10.5281/zenodo.237643 ( Johnstone *et al.*, 2016b)

License: BSD 3-Clause

The code contains the experimental input data required to reproduce the examples shown here in comma separated value (CSV) format in the file data/crumb_data.csv. Installation instructions for the tool and its dependencies can be found in the README file, in the main folder at the above links.
