# Supplementary material for: Hierarchical Bayesian inference for ion channel screening dose-response data
Source: Wellcome Open Res. 2017 Mar 13;1:6. Originally published 2016 Nov 15. [Version 2] doi: 10.12688/wellcomeopenres.9945.2 (PMC5134333; doi:10.12688/wellcomeopenres.9945.2)
Supplement: Peer review history

# Peer review: Referee response for version 1

**DOI:** 10.21956/wellcomeopenres.10720.r17624

**Published:** 2016-11-30

**Authors:** Derek Leishman

**Affiliations:** 1 Eli Lilly and Company, Indianapolis, IN, USA

This is an interesting and stimulating manuscript. The structure of the manuscript in title, abstract, problem statement (captured in the introduction and motivation), methodology, results and conclusions is appropriately clear and all sections are well written. The conclusions are well founded. The motivation in particular for the software tool is clear as is the potential impact of the tool. 

 The intended reader who might benefit most from the manuscript and tool - the experimental electrophysiologist generating ion channel screening data to predict potential safety issues - may find the methodology and underlying math daunting. Nonetheless there are principles described which need to be appreciated by those generating those data, especially when that data is further propagated in *in silico* models of cardiac tissues. The authors have written and formatted the article very effectively to make the manuscript understandable and approachable. Given the availability of computational tools such as Python it is now also much more practical to perform these analyses and appropriately quantify the uncertainty. The practice of reporting 'best-fit' parameter values may have been the best which could practically be achieved previously. It may also have been a model sufficient for many questions but the developing need to use these data in computational models of tissues for safety purposes requires a more robust treatment of uncertainty. Simply carrying forward best-fit values and generating point estimates of effects on tissues and point estimates of margins between therapeutic drug concentrations and concentrations impacting cardiac tissues may not adequately serve drug development and regulatory assessment.

 In the practice of electrophysiology screening experiments some compromises are made in order to make the experiments both quick enough and inexpensive enough to meet the drug discovery process. This will often mean that a limited range of concentrations are tested and a minimum number of individual experimental repeats are made. When calculating 'best-fit' parameters some values are 'fixed' to make calculating the key parameter IC50 possible e.g. fixing minimum current inhibition to 0%, maximum inhibition to 100% and the Hill Slope to 1. The manuscript describes some of the potential issues which may result from such compromises. There are also hidden impacts of experimental design. As an example the experiment may call for following compound effect for as long as necessary to reach a steady state effect. As the kinetics of channel block are concentration dependent this means that steady state will occur more slowly at low concentrations. In addition to drug effect there is also often current 'run-down' or 'run-up' evident in these experiments. The 'run-down' effect is the more common. This means that at low concentrations of compound the observed effect may have a larger contribution of 'run-down' than at larger concentrations. This serves to somewhat flatten the Hill Slope and left shift the IC50 from 'truth'. Alternatively, fixed durations of observation may be applied at each concentration. As the norm is to try and minimize the overall duration of the recording this may mean that compound effect is under estimated at low concentrations leading to the Hill slope becoming steeper and the IC50 being slightly right shifted. Some electrophysiologists may choose to have uniform long compound application periods or use run-down correction techniques to minimize these experimental impacts on parameter estimates. The significant value of the current manuscript is that it allows the data from the experiments to be used in their most raw form without the compromises described. It leverages priors based on previous experimental information.  It gives the key information as a probability distribution rather than a single value. It also serves to illustrate the uncertainty and give the opportunity to generate more data for those compounds which show promise and where more certainty is desirable.

 There is one key outstanding element which the current manuscript cannot address. In this experimental context it is the assessment of the kinetics of block. When relating the effect of a compound to how it may impact a tissue when the underlying kinetics of the ion channel experiment and that of the ionic current in a tissue under the normal heart rates differ cannot be captured in an IC50 value or Hill slope even taking into account the uncertainty. This will likely require different experimental voltage-clamp paradigms and the estimation of at least one further parameter. That said that parameter estimate then becomes accessible to the types of calculation described here. 

 Overall this is a very well thought out piece of work, well written and with a potentially useful piece of software described.

 This review is based on my personal professional opinion.

I have read this submission. I believe that I have an appropriate level of expertise to confirm that it is of an acceptable scientific standard.

# Peer review: Referee response for version 1

**DOI:** 10.21956/wellcomeopenres.10720.r17622

**Published:** 2016-11-28

**Authors:** Jonathan Stott, Stanley Lazic

**Affiliations:** 1 Quantitative Biology, IMED Discovery Sciences, AstraZeneca, Cambridge, UK

**Summary of the Paper**

 In this manuscript Johnstone *et al *propose a new tool (PyHillFit) for fitting dose response curves from cardiac ion channels. This tool uses Bayesian inference methods to determine the distributions of the parameters. They investigate two alternative models, one that ignores inter-experimental variability and a second hierarchical model that characterises variability. Using a recently published dataset of inhibition of multiple ion channels they demonstrate the use of the tool and how experimental variability propagates through to the fitted dose response curves. They then extend these to simulations using a cardiac action potential model to show the distribution of model outputs.

 The benefits of a hierarchical Bayesian approach are clearly demonstrated. The paper describes the tool and its underlying model and assumptions well, but could benefit from further development of the arguments around the cardiac action potential distribution.

**Main Comments**

 The most interesting part of the paper was propagating the uncertainty though the action potential simulations. The authors conclude that this measure is insufficient to distinguish compounds in terms of risk (Fig 15), but if the distributions were plotted so that they are not on top of each other it may indeed be possible to distinguish the compounds. For example, "caterpillar plots", boxplots, or violin plots (e.g. Fig 2 in http://journals.plos.org/plosone/article?id=10.1371/journal.pone.0096431) would better show the result.

 The authors also do not attempt to classify the compounds on the basis of their distribution (e.g. >50% of the distribution over a 10% prolongation of APD(90) to indicate risk of TdP) and so demonstrate how poor (or good) the distributions are as a predictive tool. The manuscript would be strengthened with this additional information.

**Minor Comments**

 It is unclear why the PyHillFit package was developed as these models can be easily fit with existing MCMC software such as JAGS or Stan. Furthermore, using existing MCMC software allows other models to be fit (e.g. 4 parameter logistic models -- when the upper and lower asymptote are not rescaled to 100% and 0%), and the sampling may be more efficient (especially with Stan, which handles hierarchical models well). The benefits of PyHillFit over these other tools should be highlighted.

 The data are analysed with two models but the verbal description of the models is ambiguous. There are three models that could be run:

1. Parameters (IC50 and Hill) are common for all experiments (complete pooling, corresponding to the first model)
2. Parameters are different for all experiments; each experiment has its own parameter estimated independently from the other experiments (no pooling; not used in this paper)
3. Parameters are different for all experiments, but shared across experiments and thus mutually informative (partial pooling; the second model)

It is clear from the equations that their second model is number 3 above, but the verbal description is that of number 2. Please clarify the verbal description.

 Other minor suggestions and corrections

- Figures 2 and 7 should have a shaded "x" node pointing into "y", as y depends not just on parameters but on other observed data.
- Figure 2, and Eq 7. Is this across all compounds and channels or separately for each compound and channel combination?
- Figure 10 might benefit from a line showing the prior distributions to allow easy comparison of prior and posterior.
- Figures 12 (A-C), 13 (A-C), 14 (A-C) would be clearer if the data points were plotted above the distribution curves
- Not clear how many cardiac action potential simulations are used to generate the distributions for Figure 15. Surely not the 375,000 samples from the posterior?
- Author contributions: RJH should be RHJ?

We have read this submission. We believe that we have an appropriate level of expertise to confirm that it is of an acceptable scientific standard.

# Response

**Published:** 2017-02-27

**Authors:** Gary Mirams

We would like to thank the reviewers for their thoughtful, insightful and careful review, we have uploaded a second version of the manuscript which we hope is improved in light of their comments.

*> The most interesting part of the paper was propagating the uncertainty though the action potential simulations. The authors conclude that this measure is insufficient to distinguish compounds in terms of risk (Fig 15), but if the distributions were plotted so that they are not on top of each other it may indeed be possible to distinguish the compounds. For example, "caterpillar plots", boxplots, or violin plots (e.g. Fig 2 in http://journals.plos.org/plosone/article?id=10.1371/journal.pone.0096431) would better show the result.*

- Thank you for the suggestion, we have updated Figure 15 to use a violin plot. This does allow a better inspection of the distributions and their overlap, as we can now see more clearly, there is still substantial overlap.

*> The authors also do not attempt to classify the compounds on the basis of their distribution (e.g. >50% of the distribution over a 10% prolongation of APD(90) to indicate risk of TdP) and so demonstrate how poor (or good) the distributions are as a predictive tool. The manuscript would be strengthened with this additional information.*

- This is something we specifically tried to avoid, partly because it becomes a large exercise that detracts from the main software tool, and also not least because the risk of TdP is not well-defined or agreed upon for some of these compounds. Our point isn't that the distributions will help/hinder predictions per-se, rather you could easily be anywhere 'within' a distribution, so wherever they overlap there will necessarily be no way to reliably distinguish between compounds. If the 50% value happened to be a good classifier for these data, we would still not expect this to hold for new compounds, as the ordering could easily reverse when new data become available wherever the distributions overlap to this extent. Nevertheless, we annotated Figure 15 with risk classifications from the CredibleMeds database (a more credible data source than its name suggests!). As one might expect, only the clear shorteners and prolongers correspond well to ‘no known risk’ or ‘known TdP risk’; the majority of compounds have overlapping distributions of moderate APD prolongation, that cannot really be ranked reliably.

*> It is unclear why the PyHillFit package was developed as these models can be easily fit with existing MCMC software such as JAGS or Stan. Furthermore, using existing MCMC software allows other models to be fit (e.g. 4 parameter logistic models -- when the upper and lower asymptote are not rescaled to 100% and 0%), and the sampling may be more efficient (especially with Stan, which handles hierarchical models well). The benefits of PyHillFit over these other tools should be highlighted.*

- PyHillFit is intended for the specific case of ion channel screening data. We do appreciate that this could have been coded in Stan, but we wanted to have more ‘control’ over what was happening below the surface, and in how the results were outputted and analysed, so we wrote the algorithms ourselves in Python. Perhaps as much of our ‘work’ here was in deriving the statistical model and appropriate prior distributions, as it was in writing the software itself. If a 4 parameter agonist compound was identified then an extension in Stan to cover this might be appropriate.

*> The data are analysed with two models but the verbal description of the models is ambiguous...it is clear from the equations that their second model is number 3 above, but the verbal description is that of number 2. Please clarify the verbal description.*

- Text amended in section 4.1.

*> Other minor suggestions and corrections*

- We appreciate these being pointed out and have made the suggested amendments:

*>    Figures 2 and 7 should have a shaded "x" node pointing into "y", as y depends not just on parameters but on other observed data.*

- Updated, thanks for the suggestion.

*>    Figure 2, and Eq 7. Is this across all compounds and channels or separately for each compound and channel combination?*

- Separately, text updated.

*>    Figure 10 might benefit from a line showing the prior distributions to allow easy comparison of prior and posterior.*

- Updated, thanks for the suggestion.

*>    Figures 12 (A-C), 13 (A-C), 14 (A-C) would be clearer if the data points were plotted above the distribution curves*

- Updated, thanks for the suggestion.

*>    Not clear how many cardiac action potential simulations are used to generate the distributions for Figure 15. Surely not the 375,000 samples from the posterior?*

- Now mentioned in the caption.

*>    Author contributions: RJH should be RHJ?*

- Updated(!), well spotted!
